# Mouse Models of Germinal Center Derived B-Cell Lymphomas

**DOI:** 10.3389/fimmu.2021.710711

**Published:** 2021-08-12

**Authors:** Stefanie N. Meyer, Sanjay Koul, Laura Pasqualucci

**Affiliations:** ^1^Institute for Cancer Genetics, Columbia University, New York, NY, United States; ^2^Department of Biological Sciences & Geology, Queensborough Community College (City University of New York), Bayside, NY, United States; ^3^Department of Pathology & Cell Biology, Columbia University, New York, NY, United States; ^4^The Herbert Irving Comprehensive Cancer Center, Columbia University, New York, NY, United States

**Keywords:** germinal center, lymphoma, genetics, mouse models, transgenic

## Abstract

Over the last decades, the revolution in DNA sequencing has changed the way we understand the genetics and biology of B-cell lymphomas by uncovering a large number of recurrently mutated genes, whose aberrant function is likely to play an important role in the initiation and/or maintenance of these cancers. Dissecting how the involved genes contribute to the physiology and pathology of germinal center (GC) B cells –the origin of most B-cell lymphomas– will be key to advance our ability to diagnose and treat these patients. Genetically engineered mouse models (GEMM) that faithfully recapitulate lymphoma-associated genetic alterations offer a valuable platform to investigate the pathogenic roles of candidate oncogenes and tumor suppressors *in vivo*, and to pre-clinically develop new therapeutic principles in the context of an intact tumor immune microenvironment. In this review, we provide a summary of state-of-the art GEMMs obtained by accurately modelling the most common genetic alterations found in human GC B cell malignancies, with a focus on Burkitt lymphoma, follicular lymphoma, and diffuse large B-cell lymphoma, and we discuss how lessons learned from these models can help guide the design of novel therapeutic approaches for this disease.

## Introduction

B-cell lymphomas are a spectrum of genetically, phenotypically and clinically diverse neoplasms that arise from the oncogenic transformation of B cells at various developmental stages and, in most cases, from germinal center (GC) B cells (1–3). Over the past two decades, studies aimed at charting the genetic landscape of these malignancies have uncovered a large number of recurrently mutated genes with potential pathogenic roles in these diseases (4–8). In order to understand the mechanisms by which these alterations contribute to lymphomagenesis, genetically engineered mouse models (GEMMs) have proven and will likely continue to prove instrumental, particularly in the case of GC-derived lymphomas, as an *in-vitro* system that faithfully recapitulates the complex biology of the GC reaction is still lacking. By mimicking genetic alterations that are found in the human disease, these models have allowed the detailed *in-vivo* investigation of several lymphoma-associated oncogenes and tumor suppressors, shedding light on their role in normal B cell development and tumorigenesis. It has to be said that each of the approaches used present specific advantages and disadvantages; for instance, GEMMs cannot reproduce the genetic complexity and the heterogeneity of the human tumors, an aspect especially important when aiming at the discovery and pre-clinical testing of novel therapeutics. To overcome this hurdle, patient-derived xenografts have been introduced for the validation of candidate biomarkers and molecular targets (9–11). Moreover, the versatility of the clustered regularly interspersed short palindromic repeats (CRISPR)-Cas9 technology and its high efficiency for precise genome manipulation in mouse embryonic stem (ES) cells has opened the way to the construction of a new array of *in vivo* experimental models. Although no single model can individualy address the wide range of questions that remain to be investigated, access to the appropriate *in vivo* tools will greatly benefit the lymphoma community. In this review, we summarize the insights gained from modeling recurrent genetic lesions associated with human B cell malignancies, focusing on three common GC-derived non-Hodgkin lymphomas for which GEMMs that faithfully recapitulate key aspects of the human disease have been achieved: Burkitt lymphoma (BL), follicular lymphoma (FL), and diffuse large B-cell lymphoma (DLBCL). We refer the reader to the work of Huang and Yasuda for an overview on mouse models of EBV-driven lymphomas (12).

## Germinal Centers: The Origin of Most B-Cell Lymphomas

The development of mouse models that recapitulate with fidelity the human disease is intimately linked to a deep understanding of the pathogenesis of these tumors and particularly of their normal cellular counterpart, as the genetic lesion of interest should be targeted to the proper temporal and developmental stage context. For most B-cell lymphomas, this is represented by a GC B cell, as documented in the nineties by the analysis of clonally rearranged immunoglobulin genes in various lymphoma subtypes (1, 2). These studies invariably showed that BL, FL and DLBCL exhibit the imprinting of somatic hypermutation (SHM), an irreversible marker of GC transit. Thus, although the tumorigenesis process may be initiated at earlier stages of B cell differentiation (see the occurrence of BCL2 translocations in FL and DLBCL), the “tumor precursor cell” undergoes its final clonal expansion in the GC.

**Figure 1:**
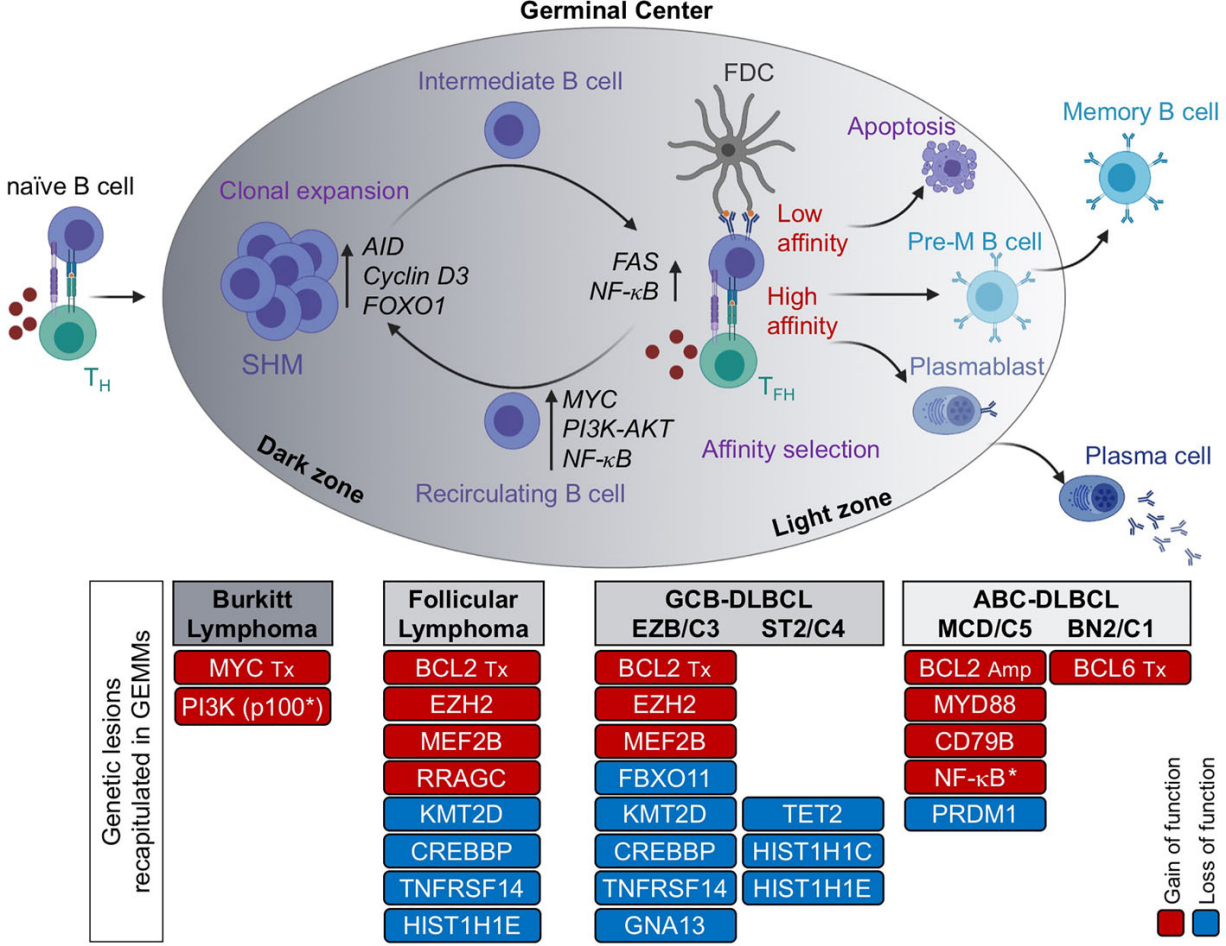
The GC reaction as the normal counterpart of most B cell lymphomas. Formation of a GC begins when a naïve B cell encounters an antigen in the presence of co-stimulatory molecules, provided by a T helper cell. The GC is functionally and histologically divided into two main compartments, the dark zone (DZ) and the light zone (LZ). Within the DZ, cells undergo somatic hypermutation (SHM) of their immunoglobulin genes and rapid proliferation, whereas in the LZ, B cells are intermingled with follicular dendritic cells (FDCs) and T follicular helper (T_FH_) cells, which provide positive selection signals to B cells with high affinity to the antigen. The LZ is also the site of CSR. By repeatedly cycling between the DZ and the LZ, B cells undergo several rounds of proliferation, SHM and affinity maturation before being positively selected. In contrast, B cells with low affinity for the antigen are eliminated by apoptosis. High affinity B cells that exit the GC differentiate into long-lived memory B cells or antibody-secreting plasma cells. Based on molecular profiling, Burkitt lymphoma is postulated to derive from DZ B cells, FL and GCB-DLBCL from LZ B cells, and ABC-DLBCL from B cells poised to undergo terminal differentiation (plasmablasts) or, in a subset of cases, pre-memory B cells. Genetic lesions that have been successfully modelled in the mouse and recapitulate key features of the human disease are indicated. Red, gain of function events; blue, loss of function events. M, mutation; Tx, translocation; Amp, copy number gain/amplification; *, pathway activation by use of a constitutively active protein.

GCs are specialized structures that form transiently in secondary lymphoid organs upon encounter of a naïve B cell with its cognate antigen in the context of T cell-dependent, adaptive immune responses (13–15). The GC reaction serves one major purpose, that is to produce a population of cells capable of secreting high-affinity antibodies against the invading pathogen (i.e., plasma cells), or of maintaining the memory of that antigen for life (i.e., memory B cells), such that they can quickly differentiate into effector plasma cells upon recall responses against the same antigen (**Figure 1**) (16). Within the GC microenvironment, B cells cyclically recirculate between two anatomical areas known as the dark zone (DZ) and the light zone (LZ) (17). DZ B cells (also called centroblasts) proliferate at high rate and modify their immunoglobulin variable (*IgV*) region genes by the process of SHM, to generate antibody specificities with different affinity to the antigen. DZ B cells then cease proliferating and evolve into LZ B cells (also known as centrocytes), a more quiescent population that is again exposed to the antigen, retained on the surface of follicular dendritic cells (FDCs) in the form of immune complexes, and then compete for help by T-follicular helper (T_FH_) cells in order to receive survival signals and undergo affinity-based selection (18). GC B cells that are not positively selected because the newly introduced somatic mutations led to a decrease in affinity, disrupted the antibody structure, or generated autoreactive antibodies, are destined to die by apoptosis. A subset of LZ B cells upregulate MYC and recycle to the DZ to undergo further rounds of SHM and selection (19, 20). Eventually, high affinity LZ B cells will differentiate into antibody secreting plasma cells or memory B cells. The GC LZ also supports the process of class switch recombination (CSR), a second AID-dependent B cell-specific DNA-modification that confers distinct effector functions to antibodies with identical specificities; however, recent work provided experimental evidence that CSR takes place predominantly in the early phases following antigen encounter, prior to the GC reaction and to SHM (21).

Consistent with this functional compartmentalization, GC DZ and LZ B cells are characterized by distinct epigenetic and transcriptional profiles that sustain diverse biological programs, with proliferation and DNA replication being enriched in DZ B cells, and a variety of signaling pathways downstream of surface receptor molecules being activated in LZ B cells, including the B-cell receptor (BCR) and the CD40 receptor (22). This oversimplified view of the GC reaction has been refined to higher granularity by recent single cell analyses of gene expression and somatically mutated *IgV* region genes in human GC B cells (23, 24). These studies revealed multiple subclusters of DZ and LZ B cells, along a continuum of transcriptional changes reflected in several intermediate subpopulations that bidirectionally recirculate between the DZ and LZ compartment, ultimately giving rise to precursor memory B cells and plasma blasts (**Figure 1**).

With the advent of genome-wide expression profile technologies, numerous studies have documented the close similarity between the phenotype of normal bulk GC B cell subsets and the transcriptional signature of various lymphoma entities, allowing a more refined assignment of BL, FL and DLBCL to their putative normal cellular counterpart, as well as the identification of functionally relevant disease subtypes (25, 26). For example, BL was found to show a gene expression profile that is closely related to the GC DZ signature, indicating a cellular origin from DZ B cells that are actively undergoing SHM (22). Conversely, FL closely resembles early LZ B cells representing an intermediate GC B cell stage, although at the single cell level tumor cells feature a desynchronization of the canonical gene expression programs found in their normal counterpart (23). Finally, at least two distinct phenotypic subtypes of DLBCL have been recognized by bulk gene expression profiling based on their similarity to distinct cellular counterparts within the GC: the so-called germinal center B cell like (GCB)-DLBCL, which is transcriptionally more similar to intermediate and LZ B cells (22, 27); and the activated B cell like (ABC)-DLBCL, which resembles *in vitro* activated B cells and corresponds *in vivo* to a small subset of LZ B cells poised to undergo plasma cell differentiation (27), but also includes, as recently suggested, cases with similarities to memory B cells (24, 28). The clinical relevance of the “cell-of-origin” (COO) classification is underscored by the association of GCB- and ABC-DLBCL with distinct prognostic categories, which supported its incorporation into the updated WHO classification of lymphoid malignancies (3). Nonetheless, it is likely that additional subgroups exist within and across this heterogeneous disease, where as many as 20% of cases remain unclassified. Confirming this notion, different genetic subsets were recently revealed based on genetic profiles, which also display separate clinical outcomes (29–31); moreover, two clinically relevant DLBCL subgroups exhibiting particularly favorable and poor prognosis, respectively, were identified by applying a single-cell based COO classification (24), warranting additional studies aimed at dissecting the complexity of this disease.

## Types of GEMMs

**Figure 2:**
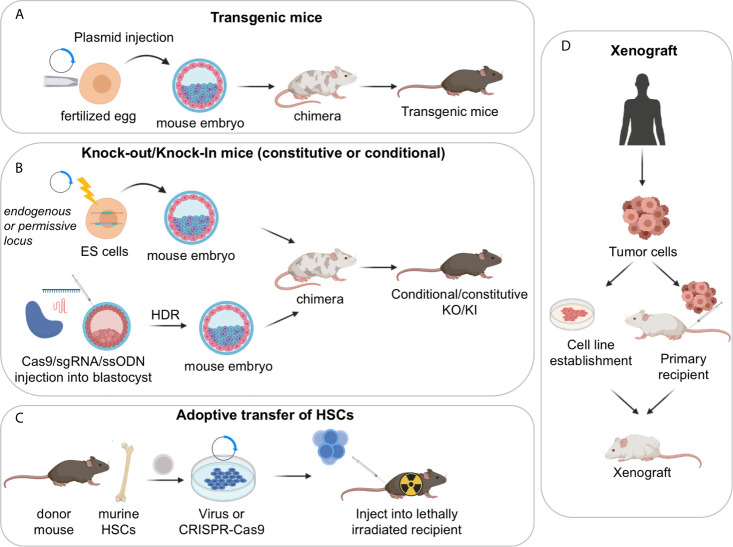
Approaches used to generate mouse models of GC derived lymphomas. **(A)** Transgenic mouse models; **(B)** constitutive or conditional knock-out/knock-in mouse models, obtained *via* homologous recombination in ES cells or CRISPR-Cas9 mediated genome editing; **(C)** adoptive transfer of HSCs. **(D)** Xenograft mouse models of human lymphoma cells can be established by injection of stable cell lines or by direct implantation of primary tumor samples into recipient immunosuppressed or humanized mice. Serial passaging of the engrafted tumors may be necessary to achieve high xenotransplantation efficiency. These models are particularly useful for the pre-clinical evaluation of novel therapeutic combinations *in vivo*.

GEMMs represent a powerful tool for the study of human cancers as well as non-malignant diseases, because over 90% of the mouse and human genomes share regions of conserved synteny, and mouse ES cells are amenable to be genetically manipulated, allowing the construction of the mutation of interest in the context of an immune system that is comparable to the human counterpart. In the B-cell lymphoma field, a variety of mouse models have been generated for the overexpression or deletion of oncogenes and tumor-suppressor genes that are linked to the human condition, by using the following main strategies: i) classical transgenic approaches, ii) targeted approaches based on homologous recombination in ES cells (i.e., knock-in/knock-out mouse models, either constitutive or conditional); and iii) adoptive transfer of manipulated hematopoietic stem cells (HSC) (**Figures 2A–C**) (32–34). More recently, the development of the CRISPR-Cas9 system, a genome-editing tool for efficient and precise genome engineering, has begun to transform the field by allowing to create virtually any mutation, thus expanding our possibilities to generate elaborate mouse-models.

The simplest *Transgenic Mouse Models* are obtained by random integration of a DNA construct into the genome upon injection into the pronucleus of fertilized eggs. These earlier models have provided critical information about the function of specific genes; however, transgenic approaches do not allow the control of the transgene copy number nor its integration site/s, which can be biased. Moreover, only a limited number of endogenous promoters are available to ensure the proper spatial and temporal control of gene expression. As such, most classical transgenic mouse models did not accurately mimic the type and/or the timing of the genetic lesion of interest, resulting in the development of tumors that do not always recapitulate the biology of the human disease. Accordingly, the field is moving away from using these lines, with few exceptions (e.g. the *VavP-Bcl2* and *Bcl2-Ig* mice discussed in the Follicular Lymphoma section) (35, 36).

*Constitutive Knock-in/knock-out Mouse Models* leverage on homologous recombination to modify endogenous genomic loci and introduce activating mutations in proto-oncogenes, disrupt tumor suppressor genes, or place a mutant cDNA under the control of a highly expressed heterologous promoter/enhancer element hijacked by chromosomal translocations in the human tumors (typically, the immunoglobulin genes). A successful example of the latter approach is represented by the Iµ-HABCL6 mouse model, where a BCL6 cDNA cassette was targeted downstream the endogenous immunoglobulin Iµ promoter to generate a chimeric transcriptional unit reproducing the outcome of a common *BCL6* chromosomal translocation variant found in DLBCL (37) (further discussed in the DLBCL section).

**Figure 3:**
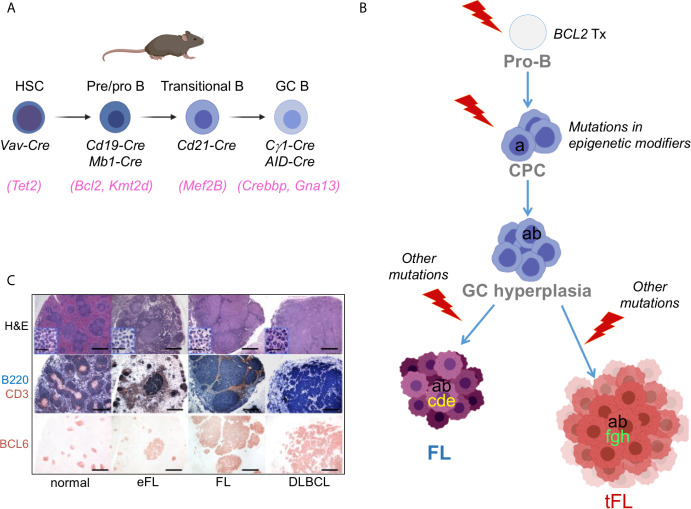
Benchmarking GC-derived GEMMs. **(A)** Cre-drivers utilized to achieve deletion/mutation of lymphoma-associated genes at the appropriate stage of B cell development, namely a HSC (e.g. *TET2* mutations), an early B cell (e.g. *BCL2* translocations), a transitional/mature B cell (utilized for genes whose endogenous promoter is specifically induced in the GC, e.g. *MEF2B*), or a GC-B cell (e.g. *CREBBP/KMT2D*). **(B)** Divergent evolution model for the pathogenesis of FL and tFL inspiring the construction of compound GEMMs. The original B cell clone is on top; based on genetic evidence, *BCL2* translocations are thought to represent the earliest event, which takes place in a pro-B cell as a by-product of the VDJ recombination process. Subsequent gain of *CREBBP* and *KMT2D* mutations by a postulated common mutated precursor cell primes epigenetic reprogramming, favouring its persistence for years before the independent acquisition of distinct genetic alterations leads to final clonal expansion and malignant transformation into a FL or a tFL, through branching evolution. **(C)** Representative histo-pathological and immuno-phenotypic characterization of lymphoproliferative diseases developing in GEMMS of lymphoma [from (41)].

*Conditional Knock-in/Knock-out Mouse Models.* The generation of mouse strains where the Cre-recombinase enzyme is expressed under the control of spatially and temporally controlled promoters has greatly advanced our ability to construct faithful mouse models by directing the introduction of candidate mutations to various stages of B cell differentiation, thus allowing the conditional activation (via removal of a *loxP*-flanked “stop cassette”) or inactivation of specific genes in the desired cell type. For instance, the crossing of floxed alleles to *mb1-Cre* (38) or *Cd19-Cre* (39) deleter strains permits gene recombination at early B-cell developmental stages and therefore throughout B cell development, whereas the *Cd21-Cre* recombinase is specifically active in peripheral B cells, from the transitional B cell stage (40). By far the most relevant Cre-recombinase alleles for the design of BL, FL and DLBCL mouse models are the *C*γ*1-Cre* and *Aicda-Cre* knock-in alleles, which allow for precise Cre-mediated gene recombination in antigen-activated mature B cells, including GC B cells (**Figure 3A**) (42–44). Conditional knock-out alleles have been successfully employed to study the *in vivo* role of many lymphoma-associated tumor suppressor genes encoding for transcription factors (BLIMP1), epigenetic modifiers (EZH2, CREBBP, KMT2D, TET2), small G proteins (GNA13) and ubiquitin ligases (FBXO11). Likewise, conditional Cre-mediated activation of mutant gain-of-function alleles, under the control of the gene endogenous promoter (MEF2B-D83V) or in the context of the permissive ROSA26 locus (BCL2), allowed to investigate their contribution to tumor development *in vivo*.

*Adoptive transfer approaches* involve the isolation of hematopoietic progenitor cells (HPCs) from the bone marrow (BM) or fetal liver of a donor mouse and their subsequent genetic modification using retroviral vectors or the CRISPR-Cas9 tool, prior to BM transplantation into recipient animals. Typically, short-hairpin RNAs (shRNAs) are used for loss-of-function studies and cDNA cassettes are used to reproduce gain-of-function mutations, whereas the CRISPR-Cas9 editing approach can serve both purposes. These modified progenitor cells will then reconstitute the hematopoietic system of lethally or sub-lethally irradiated syngeneic animals, resulting in chimeric mice with a hematopoietic system derived from the donor cells (45). The adoptive transfer approach offers the advantage of being highly versatile and rapid, without the need for breeding with additional transgenic animals (34). It has however limits in the duration of the animal follow up and the potential effects of host irradiation. Mouse models obtained using this technique demonstrated that reduced dosage of *Kmt2d* or *Crebbp* accelerates Bcl2-driven lymphomagenesis by affecting the deposition of activating histone marks onto the regulatory domains of genes implicated in GC exit (discussed in the FL section) (46, 47).

*CRISPR-Cas9 editing approaches.* The advent of the CRISPR-Cas9 technology has transformed the approach to genome editing, as the Cas9 nuclease can be targeted to any specific, 20 nucleotide-long genomic sequence that is followed by a protospacer-adjacent motif (PAM), where it will cut the DNA (48). These DNA breaks can then be repaired by non-homologous end joining, leading to small insertions/deletions, or by homology-directed repair (HDR), which can be leveraged to generate precise DNA modifications by providing a DNA template. HDR can introduce point mutations, insertions of DNA sequences (e.g. protein tags, *LoxP* sites) or specific deletions. This approach, which is extensively reviewed elsewhere (49–51), bears the advantage of being rapid while maintaining the endogenous regulation of expression of the gene studied, and can allow complex manipulations involving multiple independently segregating alleles (52), although the construction of conditional alleles has been challenging. In the context of B cell lymphomas, a CRISPR-Cas9 based design was successfully utilized to engineer two activating mutations in the gene *RRAGC*, recurrently detected in FL patients (53) (see FL section). A lists of GEMMs recpitulating GC-derived lymphomas is reported in **Supplementary Table 1**.

## Patient-Derived Xenograft Models

Despite many advantages offered by GEMMs in understanding the basic mechanisms underpinning tumor development, conditional transgenic mice present some limits particularly in the context of preclinical oncology. First, they are expensive to generate, involve laborious techniques, and may require long time to establish a large animal cohort of the desired genotype, because many littermates will not carry the desired combination of alleles after crossing. Moreover, GEMMs are generated from inbred mouse strains and model only few mutations at a time; as a consequence, tumors developing in these animals may not recapitulate the genomic complexity of human lymphomas, and could thus be less clinically relevant. Finally, the variable onset and penetrance of disease makes them suboptimal models for drug development and testing.

To circumvent some of these problems, efforts have been made to directly implant tumor tissues or cells surgically dissected from cancer patients into immune-compromised recipient mice by subcutaneous, intravenous or orthotopic transplantation (**Figure 2D**). When successful, such model system, known as Patient Derived Tumor Xenografts (PDX), was shown to maintain the same genetic and histopathologic characteristics of the original tumor clone, and thus to better represent the genomic complexity of the human disease, in a way that is hard to achieve in GEMM (54, 55). However, more recent work has indicated that PDX models rapidly acquire copy number aberrations during passaging, most likely due to the expansion of minor clones present in the parental tumor, which could raise concerns about their role in cancer studies (56, 57). PDXs can be propagated without *in vitro* manipulation and have been used in several preclinical studies aimed at confirming findings obtained in *in vitro* cell lines and/or at assessing drug responses. Nonetheless, testing of multiple PDX models is necessary in order to obtain generalizable results, which quickly increases the complexity of the experiments. PDXs also require significant infrastructural support and may take several months before engraftment is achieved, which is why large repositories such as the Public Repository of Xenografts (ProXe) database or the Novartis Institutes for BioMedical Research PDX encyclopedia (NIBR PDXE) have been generated (10, 58). The establishment of several DLBCL and transformed FL PDXs has been reported, which can be stably propagated *in vivo* and reflect phenotypic and genetic features of the GCB- and ABC-DLBCL subtypes, while maintaining key genetic drivers of pathogenesis that were present at diagnosis (10, 59).

The major disadvantages of xenograft models are the lack of a physiological tumor microenvironment (unless in the context of orthotopic injections) and the lack of a functional immune response. The injection of tumor cells into the tissue of origin more closely mimics microenvironmental cues provided by the non-neoplastic cells, allowing the interaction between these components, even though differences in signaling pathways or cellular populations might be expected between the human and mouse microenvironment. The lack of a functional immune response can be partially addressed in NOD/SCID mice by addition of human peripheral blood lymphocytes, bone marrow, or fetal liver and thymus into irradiated or immunodeficient mice (60). However, due to the development of graft versus host disease, the observational window in these humanized mice is relatively short (61). Despite these issues, PDX models are expected to provide an improved platform for testing drug sensitivities and investigating the development of drug resistance, as well as for the validation of biomarkers (54), particularly when compared to cell line derived xenografts (62–65).

## Mouse Models of Burkitt Lymphoma

The genetic hallmark of BL is a chromosomal translocation that brings the *MYC* gene under the control of one of the *IG* enhancers (66, 67), causing its ectopic transcription in the bulk GC population where MYC expression is otherwise limited to a small subset of cells primed for DZ re-entry (19, 20). Additionally, several genes were identified as recurrently mutated in this lymphoma. These include *ID3*, a negative regulator of TCF3/E2A that is inactivated in 35-58% of all BL subtypes, and *TCF3*, which encodes the transcription factor E2A and is targeted by gain-of-function mutations in 10-25% of cases (68–70). The TCF3-ID3 axis is predicted to promote antigen independent “tonic” BCR signaling, leading to the sustained activation of the phosphoinositide-3-kinase (PI3K) signaling pathway and therefore providing pro-survival signals to the tumor cell. Gain-of-function mutations of *CCND3* (5% of endemic BL and 38% of sporadic BL), which encodes for a D-type cyclin required for the proliferation of DZ B cells, and missense mutations of the FOXO1 transcription factor (20% of cases) are also recurrently found in different clinical variants of BL, highlighting a prominent oncogenic role for these two genes (69–73).

### Mouse Models of Deregulated MYC Expression

As the first oncogenic translocation identified in B-cell lymphomas, several transgenic mouse models have been generated over the years to drive **MYC** overexpression throughout B-cell development under the control of different *IG* enhancers, in an attempt to mimic the *IGH-MYC* translocation (74–77). At the time, it was not known that these lesions occur as by-products of the SHM or CSR process, that is, during the GC reaction. As a result of such early activation, most MYC-transgenic models develop pre-GC derived B-cell lymphomas that, while reproducing some histo-morphologic features of the human disease, lack surface *IG* expression (EµMYC mouse model) (74, 78) or retain transitional B cell markers (e.g. CD43) in the absence of somatically mutated *IGHV* regions (λ-MYC mouse model and 3’ IgH LCR-driven Myc transgenics) (75, 77), an indication that the malignant transformation process occurred in transitional/pre-GC cells. Although these models have helped to investigate the role MYC plays in oncogenesis overall, or to elucidate the cooperativity among diverse oncogenes (79–82), they are not considered informative for dissecting the pathogenesis of BL; moreover, the nearly full penetrance of immature B-cell lymphomas in some of these models may complicate the study of the GC B cell response, as mice frequently die before becoming immunologically mature.

A mouse model that mimics all key aspects of BL was generated in the laboratory of Klaus Rajewsky in 2012 (83). This was achieved by inducing the overexpression of MYC specifically in GC B cells, in combination with a constitutive active form of the PI3K catalytic subunit (referred to as mutant P110*). Tumors developing in these mice closely resemble the human BL morphologically and histologically, as well as in their transcriptional profile, including the expression of BCL6. In addition, tertiary transforming events, such as mutations in *CCND3* and ongoing SHM, were observed in the developing tumors. Thus, the Myc/P110* animal model could represent a valuable system to study the mechanisms underlying BL development, as well as the potential preclinical utility of targeted therapeutics. Using these mice, a pro-proliferative and anti-apoptotic function of FOXO1 was uncovered, which contributes to the transformation of GC B cells towards BL (84).

## Mouse Models of Follicular Lymphoma and GCB-DLBCL

FL is the second most common type of B-cell lymphoma (3). While typically an indolent disease, FL represents a continuing challenge for researchers and clinicians because it remains incurable. Moreover, a significant fraction of patients progress early or undergo histologic transformation to a more aggressive DLBCL, with poor long-term outcome (85, 86). A distinctive feature of this disease is the constitutive expression of the anti-apoptotic protein BCL2, due to the hallmark t(14;18) translocation that places the *BCL2* coding region under the control of the *IGH* enhancer (87). This genetic lesion is insufficient alone to drive lymphomagenesis in humans, as documented by the fact that *BCL2* translocations can be detected, at extremely low frequency, in the peripheral blood of most healthy individuals (88), yet the majority of these subjects will never develop a FL (89, 90). Thus, additional oncogenic events are required for the malignant transformation of these precursor cells. Indeed, whole exome sequencing analysis of large FL datasets revealed a plethora of additional, highly recurrent somatic mutations, with the majority of them targeting histone/chromatin modifying enzymes. These include the KMT2D methyltransferase, mutated in 70-80% of cases, the CREBBP acetyltransferase (65% of cases), and the EZH2 methyltransferase (22% of cases), but also multiple linker-histone family members (over 44% of cases) and, less commonly, the chromatin remodeler ARID1A (91–95). The nearly universal involvement of these genes in FL established aberrant epigenetic regulation as a central driving force in this lymphoma type, in addition to BCL2 deregulation.

Other common genetic alterations that have been successfully modeled in mice include gain-of-function mutations of *MEF2B* (15% of cases) (96), biallelic loss-of-function mutations and deletions of *TNFRSF14* (up to 40% of cases) (94, 97), and point mutations of the *RRAGC* gene. Of note, these same genes (with the exception of *RRAGC*) are also recurrently mutated in GCB-DLBCLs, and particularly in the recently identified EZB (for EZH2-BCL2)/C3 (Cluster 3) genetic subtype (29, 30). Accordingly, mouse models recapitulating these lesions develop both FL and DLBCL. We discuss them in this section because of the higher prevalence of these alterations in FL as compared to DLBCL, and the preferential development of FL-like diseases, with a smaller number of overt large B-cell lymphomas.

### Mouse Models Engineered to Mimic the BCL2 Translocation

In order to study the impact of deregulated BCL2 expression *in vivo*, several attempts have been made to genetically engineer the t(14;18) translocation in mice. Of these models, two have successfully recapitulated FL-like tumors within their lifespan: the *VavP-Bcl2* mouse model and the *BCL2-Ig* mouse model (35, 98, 99). A third model, BCL2^tracer^ mice, faithfully recapitulates the early stages of BCL2 deregulation, but does not advance to lymphomas. Similarly, Eµ-*BCL2* mice develop an expanded small B-lymphocyte population but they don’t develop tumors spontaneously (100), unless combined with other oncogenes; nonetheless, this mouse model has been useful in revealing the cooperativity between *BCL2* and other candidate oncogenic events such as *CREBBP* loss (101). More recently, mice carrying a conditional *BCL2* knock-in allele in the Rosa26 locus (Rosa26LSL.BCL2.IRES.GFP) were reported to display enlarged spleens with an increase in follicular B cells and larger GCs, when BCL2 expression was induced in pre/pro B cells using the *Cd19-Cre* deleter strain (102). These mice were designed to mimic the *BCL2* copy number gains that are frequently associated with ABC-DLBCL, rather than the t (14;18) translocation. Consistently, the B cell lymphomas developing over time in roughly 50% of these animals are largely B220^-^ and CD138^+^, indicating a post-germinal center plasmablastic differentiation. Although recapitulating a more advanced stage than that from which ABC-DLBCLs presumably derive, this background was useful to study the synergistic activity of mutations implicated in the pathogenesis of ABC-type DLBCL, and will be discussed in the DLBCL section (102).

In the ***VavP-Bcl2*** mouse model, the BCL2 oncogene was placed under the control of the pan-hematopoietic *Vav* promoter. Hence, BCL2 expression is enforced in the whole hematopoietic lineage, at an earlier developmental stage than when the human BCL2 translocation occurs (36). Despite this limitation, young *VavP-Bcl2* mice display spontaneous, antigen independent GC hyperplasia, and develop over time B-cell lymphomas that faithfully recapitulate the GC origin of the human FL, along with other critical aspects of its pathobiology such as the follicular pattern, the expression of peanut-agglutinin (PNA) and BCL6 in the absence of post-GC markers, and the presence of clonally rearranged *IGHV* genes that are somatically mutated (98). The *VavP-BCL2* model has served as an excellent experimental system for deciphering the cooperative role of other genetic lesions observed in the human condition concomitantly with *BCL2* translocations. To this end, *VavP-BCL2* mice were crossed with other GEMMs (e.g. *Crebbp^fl/fl^*, *Kmt2d^fl/fl^*, *Ezh2^Y641N^*) or were used directly as a source of HPCs that were transduced with retroviral constructs carrying gain- or loss-of-function mutants before transplantation into irradiated mice (103). Nonetheless, the ubiquitous expression of BCL2 in the entire hematopoietic lineage and the dependency of *VavP-BCL2* GC B cells on BCL2-expressing CD4^+^ T_FH_ cells could represent a drawback that investigators should carefully consider depending on the specific questions they wish to address.

Unlike the *VavP-Bcl2* mouse model, the ***BCL2-Ig*** model expresses a *Bcl2* minigene under the control of *IG* regulatory elements, and thus exclusively in B cells (35). This strain displays an excess of B lymphocytes (both small B cells and plasma cells) that were shown to survive for a prolonged period of time under *in vitro* conditions, providing the first *in vivo* evidence for the anti-apoptotic function of BCL2, independent of proliferation (35). Bcl2-Ig transgenic animals did not develop tumors in the original 12-month follow up study (35) but, when challenged by chronic immunization with a T cell dependent antigen, they were shown to accumulate GC B cells and, in 40% of cases, to develop PAX5^+^BCL6^+^ FLs, with a smaller fraction of plasmacytoid tumors (PAX5^–^BCL6^–^IRF4^+^) (104).

Perhaps the model recapitulating with most fidelity the initial steps of FL genesis, though never progressing to overt FL, is the mosaic **BCL2^Tracer^**, where expression of a functional human *BCL2* (*hBCL2*) transgene is contingent on RAG dependent inversion of this cassette during the V(D)J recombination process (105). As such, this model mimics both the sporadic nature of the t(14;18) translocation and its induction at the appropriate developmental stage, i.e. a BM pro-/pre-B cell, as a byproduct of VDJ recombination (105, 106). In these mice, the recombination event leads to a unique coding joint; thus, the frequency of recombination can be confirmed at the genetic level by PCR and at the protein level by use of specific anti-hBCL2 antibodies. Although limited to the development of *in situ* FL, the BCL2^Tracer^ model has helped in tracking the initial events leading to the accumulation and expansion of *BCL2*-translocated B cells, paving the framework for the current model of FL ontogenesis, based on three lines of evidence. First, as in the case of human *in situ* FL, when mice were challenged by T-cell dependent antigens, hBCL2-overexpressing B cells (but not the non-rearranged B cells) were triggered to make multiple GC re-entries and spread to an advanced pre-neoplastic stage. Second, while the fraction of hBCL2^+^ cells in the naïve, GC and memory B-cell compartment was comparable upon a single immunization, their number was markedly enriched in the GC and memory B cell population, following chronic antigenic recall. Finally, hBCL2^+^ cells were able to repopulate the GCs of immunized WT mice in adoptive transfer experiments (105). Together with the observation that t(14;18)-positive cells in healthy individuals harbor somatically mutated *IGHV* region genes, these data provide a plausible explanation for the origin of FL from a recirculating memory B cell requiring multiple transits through the GC, before the acquisition of additional genetic or epigenetic perturbations ultimately drives the development of clonal tumors.

### Mouse Models Recapitulating Alterations in Histone Modification Genes

A second genetic hallmark of FL and EZB/C3 DLBCL is the presence of mutations in genes encoding histone/chromatin modifiers, collectively accounting for almost all FL cases and over 50% of DLBCL cases. These lesions constitute early events in the phylogenetic history of the disease, which in the context of FL transformation can be found in the dominant tumor clone of both the indolent FL and its transformed FL (tFL) counterpart, suggesting that they have been acquired by a putative common precursor cell (CPC), before divergent evolution and final clonal expansion (**Figure 3B**) (94, 107, 108). The exact developmental stage at which *KMT2D* and *CREBBP* mutations emerge remains to be determined; thus, hemizygous and homozygous loss of these genes has been modeled at different stages of B cell differentiation, by using Cre-drivers that are specifically active in HSC (109), early B cells (*Cd19-Cre* and *mb1-Cre*) (41, 47, 101, 110) and GC B cells (via the *C*γ*1-Cre* recombinase) (41, 110, 111).

The Complex Of Proteins Associated with Set1 (**COMPASS**) plays a pivotal role in the process of mammalian transcription through mono- and di-methylation of histone 3 lysine 4 (H3K4) at enhancer/super-enhancer regions (112). This activity is executed through its catalytic subunit **KMT2D**, which is the most commonly mutated gene in FL and EZB/C3 DLBCL. *KMT2D* mutations are mainly truncating events, with few missense mutations in the SET domain, which all impair its enzymatic function, indicating that *KMT2D* acts as tumor suppressor gene in B cells. Interestingly, when *Kmt2d* was conditionally deleted in pre-B cells, that is, at a much earlier stage than when the final malignant transformation ensues, the GC B cell population expanded significantly in response to antigenic challenge, compared to wild-type littermates (41, 47). The same phenotype, but less pronounced, was observed when *Kmt2d* was disrupted at a later stage, after the initiation of the GC reaction (41). Analogously, changes in the transcriptional profile of GC B cells from *Cd19-Cre* compound mice were more robust compared to GC B cells where deletion of *Kmt2d* was induced by *Cγ1-Cre* (41). The most prominent signature lost in *Kmt2d*-deficient GC B cells includes genes implicated in cytokine signaling, IFN responses and terminal differentiation programs. These data suggest an early role for *KMT2D* inactivation in FL, likely through epigenetic reprogramming. Consistent with this model, loss of *Kmt2d* alone in the GC was not sufficient to drive lymphomagenesis, but when combined with deregulated expression of *BCL2* (as observed in human FL and DLBCL) the two cooperate, leading to a significant increase in the percentage of *bona fide* FL and DLBCL characterized by clonally rearranged, mutated *IGHV* genes and the expression of GC-specific markers (**Figure 3C**) (41). The synergistic effect of *Kmt2d* loss and *BCL2* deregulation *in vivo* was independently confirmed in a mouse model of adoptive transfer where *Kmt2d* was knocked-down in *VavP-Bcl2* HPCs prior to reconstitution into lethally irradiated syngeneic mice (47).

Mutations inactivating the acetyltransferase ***CREBBP*** (either truncating or missense in the HAT domain) are the second most common epigenetic lesion in FL (95). Together with its paralog EP300, CREBBP belongs to the KAT3 family of histone and non-histone acetyl-transferases, which modulate transcription by acetylating H3K27 and H3K18 at gene enhancers and promoters. GEMMs mimicking the conditional loss of *Crebbp* share remarkable similarities with the *Kmt2d*-KO model, including: a) the increase in GC B cells with partially overlapping transcriptional changes; b) a more pronounced GC phenotype in *Cd19-Cre* background compared to *C*γ*1-Cre* mice; c) the inability to drive full-blown tumor formation on their own, but a strong synergistic activity with *BCL2* deregulation, leading to acceleration of lymphoma onset and increased penetrance of FL (46, 110). In human GC B cells, CREBBP binds virtually all GC-specific super-enhancers; however, not all those genes are transcriptionally affected by its loss in purified murine GC B cells, as well as in DLBCL cell lines (46, 110). This might be partly due to the compensatory activity of its paralogue EP300 and, indeed, CREBBP and EP300 are rarely concurrently and biallelically mutated, indicating that GC B cells need a certain threshold of acetyltransferase activity for their survival (111). However, *CREBBP* deletion caused focal enhancer loss of H3K27Ac and reduced expression of specific genes that are involved in GC exit, such as downstream effectors of BCR and NF-κB signaling pathways, multiple cytokines, and antigen presenting molecules, with MHC class-II genes being the most notable among them (46, 110). These findings parallel the human FL, where *CREBBP* mutations are associated with decreased MHC-II expression and reduced frequency of tumor-infiltrating T-cell subsets (108). Notably, the chromatin domains occupied and acetylated by CREBBP are direct targets of the BCL6 oncorepressor in a complex with SMRT and HDAC3 (46, 110). Additionally, CREBBP directly acetylates several proteins that are relevant to B cell lymphoma biology, including the TP53 tumor suppressor, which requires acetylation for its activity, and the BCL6 protein, which instead is functionally impaired by acetylation due to the lost interaction with co-repressor complexes (95, 113). These GEMMs were critical to document a major role for CREBBP in GC B cells by opposing the oncogenic activity of BCL6 and thereby initiating the activation of terminal differentiation/antigen presentation program as LZ B cells engage T_FH_ cells and prepare to exit the GC. Consistent with these data, *CREBBP*-mutant lymphomas show reduced expression of genes that are antagonistically regulated by the BCL6-SMRT-HDAC3 complex and become dependent on HDAC3 for their survival. Conversely, when HDAC3 activity was inhibited, histone acetylation was restored at these enhancers and lymphoma growth was suppressed both *in vitro* and *in vivo* (46, 114). These studies identified HDAC3 and EP300 as vulnerabilities of *CREBBP*-mutant cells that may lead to potential therapeutic avenues for these lymphoma entities.

**EZH2**, a histone methyltransferase, catalyzes the addition of repressive H3K27me3 marks at selected, cell-context dependent regions that, in the GC, include proliferation checkpoint genes (e.g. *CDKN1A*, *CDKN1B*) and genes involved in plasma cell differentiation (e.g. *IRF4*, *PRDM1*), creating bivalent promoters that can be rapidly re-activated when B cells receive the signal to exit the GC (115). Indeed, EZH2 is required for GC formation (115, 116). Two hotspot gain-of-function mutations, Y646F (equivalent to the mouse residue Y641) and Y646N, have been observed in human lymphomas and were modeled in the mouse to study their role in lymphomagenesis. In these animals, expression of the conditional *Ezh2*^Y641F^ allele is driven by the endogenous *Ezh2* promoter (117), whereas expression of the transgenic *Ezh2* ^Y641N^ allele is under the control of the CAG promoter (115). Both alleles, when selectively activated in the GC following the *C*γ*1-Cre-*mediated excision of a lox-stop-lox cassette, led to massive GC hyperplasia, sustained by enhanced proliferation, blockade of terminal differentiation, and increased abundance of H3K27me3 levels at the promoters of Ezh2 target genes. A key element for this phenotype is the functional cooperation between EZH2 and the BCL6/BCOR repressor complex (117). In both models, expression of the mutant *Ezh2* knock-in allele did not lead to lymphomas; however, accelerated lymphomagenesis was observed when mice were crossed with *VavP-Bcl2* transgenics or upon adoptive transfer of *VavP*-*Bcl2* BM cells transduced with *Ezh2* ^Y641F^ vectors (115, 117, 118). *Ezh2* mutations were also shown to cooperate with deregulated BCL6 expression in a compound *IµHABCL*6;*Ezh2* knock-in mouse model, giving rise to a transplantable, GC-derived DLBCL-like disease. Comparatively, *Cd19-Cre* driven expression of a mutant Ezh2 protein under the control of the endogenous promoter induced B-cell lymphomas at high penetrance, but the phenotype of these tumors (B220^+^, CD19^+^, IgM^+^, CD43^+^, CD5^+^ and Mac1^+^) is not reminiscent of the human lymphomas, reinforcing the importance of achieving precise temporal and spatial control of the target genetic lesions (119). Besides documenting the oncogenic role of EZH2 mutations, the value of the *Ezh2;C*γ*1-Cre* GEMMs is twofold: first, they revealed an additional function of Ezh2 in shaping the tumor microenvironment, providing an opportunity to study syngeneic immune responses (see following section); second, they proved to be a valuable tool for the preclinical testing of novel therapeutic approaches, as tumors developing in these mice replicate the human phenotype in several aspects related to the tumor microenvironment. In particular, they display significantly lower expression of MHC-I and MHC-II, accompanied by an immunologically cold environment with reduced T-cell infiltrate, which could be restored upon treatment with EZH2 inhibitors (118).

FL and DLBCL also feature recurrent somatic mutations in histone genes, with the **linker Histone H1 family** being most commonly affected (up to 44% of FL and 26% of GCB-DLBCL cases), and the *HIST1H1C* and *HIST1H1E* family members accounting for the majority of mutations (94, 120). The cooperative role of inactivating *H1C* and *H1E* mutations, which are often concurrently found in the same case, was recently demonstrated in a double knock-out mouse model displaying an increase in both the size and number of GC structures that form upon T-cell dependent antigenic challenge. This phenotype was linked to the evidence of chromatin decompaction specifically at target genes of stem cell factors (e.g. NANOG, SOX2, and PRC2). Thus, *H1* mutations may impair proper chromatin compartmentalization and provide a fitness advantage to mature B cells by both preventing differentiation and activating stem cell like programs, including enhanced self-renewal. In line with this hypothesis, transplantation of *VavP-Bcl2;H1c^-/+^H1e^-/+^* lymphoma cells into secondary and tertiary recipient mice yielded 100% engraftment, which was not observed with the *VavP-Bcl2* only tumors, consistent with the fact that *H1* mutant DLBCL are highly aggressive (120).

**TET2** is a dioxygenase that converts 5-methylcytosine (5mC) to 5-hydroxymethylcytosine (5hmC), 5-formylcytosine and 5-carboxylcytosine, an important step in DNA demethylation (121). Oxidation of 5mC by TET2 has also been recognized as a modulator of enhancer activity during differentiation. Compared to myeloid neoplasms (122, 123), *TET2* inactivating mutations are detected at relatively low frequencies in FL/tFL (3-10% of cases) and DLBCL (6-12% of cases) (29, 30, 107, 124). Consistent with the observation that patients with *TET2* mutated lymphomas harbor the same mutation in their HSC (125), the contribution of these alterations to lymphomagenesis was studied *in vivo* by engineering the conditional loss of *Tet2* in HSCs or at later stages of B-cell development (126). *Tet2* deficiency facilitated the expansion of GC B cells in *Vav-Cre* and *Cd19-Cre* conditional KO mice, but not when directed to the GC stage, and led to promoter hypermethylation of genes implicated in GC LZ programs, with consequent transcriptional repression. However, these abnormal cells fail to advance to clonal DLBCL. When GC-specific *Tet2* deletion was combined with *BCL6* deregulation, effacement of the splenic architecture due to enlarged follicles or diffuse lymphoid infiltrates was observed. These tumors are negative for several mature B cell markers like CD23, CD21, IgM and IgD, and will require further detailed characterization. However, this work unraveled a potential link between TET2 and CREBBP in orchestrating the transcriptional program that sustains GC exit through CREBBP-dependent acetylation and stabilization of TET2, resulting in the activation of enhancer domains (126).

Together, the above studies were critical to demonstrate how mutations in epigenetic modifier genes initiate lymphomagenesis by reprogramming the epigenome of the CPC, leading to the activation of partially overlapping biological programs that, in cooperation with *BCL2* deregulation, cause malignant transformation. Identifying the specific stage at which these mutations are introduced, and the sequence of genetic or epigenetic events that cooperate with these lesions to drive full malignant transformation remains an open question that warrants further studies. Finally, the observation that MHC-II and other surface receptor molecules are regulated by epigenetic modifier genes suggests that epigenetic dysregulation may contribute to tumor immune escape by actively influencing the microenvironment.

### Mutations Affecting the Cross-Talk With the Tumor Microenvironment

Normal GC B cell development, survival and differentiation is essentially dependent on pro survival signal transduction pathways that are engaged by the cross-talk with immune and accessory cells, including the secretion of multiple cytokines and chemokines. These micro-environmental interactions play an equally important role during FL development, as they create a permissive niche to support the malignant B cell population (127, 128). Interestingly, although LZ B cells –the normal counterpart of FL– are highly dependent on T cell help, augmenting the anti-tumor immune response by checkpoint blockade approaches has been disappointing in this disease (129). Such lack of success may be due in part to multiple genetic alterations that can affect the FL (and DLBCL) microenvironment directly and indirectly, allowing escape of immune surveillance, while creating a pre-lymphoma niche that fosters malignant transformation and growth. For instance, loss of MHC-I cell surface expression has been observed particularly during FL transformation to a more aggressive DLBCL, as the result of mutations in components of the MHC-I complex or to alterations in their transcription and transport, which may favor evasion from CD8^+^ T cell immunosurveillance (118, 130, 131). Reduced MHC-II levels are also a feature of FL and DLBCL, which seems to be enriched in cases carrying mutations of CREBBP and EZH2. Together, these findings suggest a close link between epigenetic reprogramming and immune escape in these tumors, the study of which could ideally leverage on GEMMs.

***TNFRSF14***, which encodes the HVEM receptor, is mutated or deleted in 28% FL and 9% GCB-DLBCL (132, 133). *In vivo*, loss of function studies used an shRNA-knockdown strategy in the *VavP*-*Bcl2* HPC adoptive transfer system (132). Although this approach may not fully recapitulate the exact timing at which *TNFRSF14* mutations are presumably acquired in the human tumors, these mice showed an increased penetrance of Bcl2-driven FLs upon HVEM knockdown. Moreover, only a minority of T cells were found to express the *shHvem* hairpin construct, whereas *shHvem*-expressing B-lymphoma cells were significantly enriched. Mechanistically, this model revealed that HVEM loss stimulates BCR signaling and B cell proliferation both in cell-autonomous and BTLA-dependent manner. Moreover, it demonstrated the ability of HVEM low expression to induce a tumor-supportive microenvironment through increased production of TNF-family cytokines that act as stroma-activating factors. Both murine and human *TNFRSF14*-deficient FLs show prominent lymphoid stroma activation. This research offered a new therapeutic avenue by demonstrating *in-vivo* that abnormal BCR signaling and cytokine production in FL can be normalized by injecting a soluble HVEM ectodomain protein, resulting in tumor growth delay.

As mentioned, a recent study has shown that mutant ***Ezh2*** can also affect the GC microenvironment, by attenuating the requirement of T_FH_ cells for GC B cell survival (134). In particular, single cell analysis showed an expansion of the LZ compartment that was not due to impaired differentiation, but to an increase in proliferation and a reduction in cells circulating back into the DZ. Genes downregulated in *Ezh2* mutant LZ cells are normally required for the interaction with T_FH_ cells (e.g. *Tnfrsf14*, *Cd69*, *Icos* and *Icam1*) and *Ezh2* mutant LZ cells showed impaired T_FH_ interactions, suggesting that they no longer need to compete for T cell help in order to survive and undergo selection. Instead, *Ezh2* mutant GC B cells upregulated genes involved in FDC signaling. Importantly, this study showed a significant association between *EZH2* mutated FLs and an extensive FDC network. Thus, lymphoma cells carrying *Ezh2* mutations may reprogram the GC niche to allow for their own aberrant expansion in an FDC-dependent manner, and remodel the interaction between B cells, T_FH_ and FDCs. These data also raise the possibility that one of the mechanisms underlying the activity of EZH2 inhibitors against *EZH2* mutant FLs (135) is their ability to restore proper interactions between the tumor cells and the microenvironment.

### Modeling MEF2B Activating Mutations

**MEF2B** is a transcription factor that, within the B cell lineage, is exquisitely expressed in the GC (96). MEF2B instructs the GC transcriptional program by modulating a broad set of genes that are implicated in multiple biological functions and also include the BCL6 master regulator (104). This activity is hijacked in ~15% of FL and DLBCL due to a variety of somatic mutations that can be broadly classified into two groups: i) missense mutations in the protein amino-terminal portion, encoding the DNA-binding domain; and ii) truncating and missense mutations in the protein C-terminal portion, where post-translational modifications like sumoylation and phosphorylation have been mapped. While the consequences of the C-terminal group of mutations remain to be studied, the N-terminal mutations were found to prevent the physical interaction of MEF2B with components of the HUCA complex and HDAC genes, thus interfering with negative regulatory mechanisms of its activity. As MEF2B transcription is induced in the early stages of GC commitment, the role of the most common D83V N-terminal mutation was investigated in a conditional knock-in mouse model crossed with *Cd21-Cre* mice (104). *Mef2b* ^+/D83V^; *Cd21-Cre* mice display benchmark characteristics of GC-derived lymphomas, including a significantly enhanced GC response compared to their control littermates and the development of clonal FL and DLBCL in 20% of the animals, which became fully penetrant when mice were crossed with the *BCL2*-*Ig* allele.

### Modeling Metabolic Reprogramming by *RRAGC* Mutations

***RRAGC*** encodes a GTPase (RagC) involved in the activation of mammalian target of rapamycin complex 1 (mTORC1) that is responsible for the sensing and response to amino acid availability (136). Together with other components of this super-complex, *RRAGC* is mutated in ~17% of FL cases, implying an important pathogenetic role (137). A mouse model for the most common FL-associated *RRAGC* mutations was recently constructed by taking advantage of the CRISPR-Cas9 genome engineering technology to introduce the S74C and T89N sequence changes in the endogenous locus, followed by crossing with *VavP*-*Bcl2*-transgenic mice. These studies revealed that *Rragc*-mutant B cells show partial insensitivity to nutrient withdrawal, leading to accelerated FL tumorigenesis (53). The phenotype of *Rragc* mutant cells was not due to enhanced proliferation, but to reduced apoptosis, and was dependent on micro-environmental pro-survival signals normally provided by T_FH_ cells. Expression of the Rragc S74C and T89N protein increased GC B cell fitness by inducing mild activation of the mTORC1 pathway, consistent with a model whereby the mutation provides a competitive advantage to pre-malignant GC B cells, allowing them to undergo continuous cycles of selection and proliferation within the GC. This in turn could facilitate the acquisition of additional genetic alterations, and ultimately transformation into a *bone fide* FL. Interestingly, while mutations in *TNFRSF14* increased T_FH_ infiltration, *RRAGC* mutations decreased the GC dependency on T_FH_ signaling. Consistent with these opposing effects on the microenvironment, mutations in *RRAGC* and *TNFRSF14* are mutually exclusive. Targeting Rag GTPase signaling could thus represent a promising strategy against FL, warranting further efforts toward the development of specific inhibitors of nutrient signaling.

## Mouse Models of Diffuse Large B-Cell Lymphoma

DLBCL, the most common type of lymphoma in adulthood, is a heterogeneous disease comprising a diverse group of phenotypically and molecularly distinct entities associated with different clinical responses to currently available first-line chemo-immunotherapeutic approaches (4). In addition to the phenotypic classification into GCB-DLBCL and ABC-DLBCL, as many as 8 distinct genetic subgroups have been recently identified based on the co-occurrence of specific mutational events (29–31). Among these, the EZB genetic subtype and the partially overlapping C3 DLBCL share significant similarities with FL in terms of mutational profile, as reviewed in the previous section. *MYC* translocations can also be found in ~12% of tumors with DLBCL morphology, generally in the GCB type and largely in the presence of concurrent BCL2 rearrangements (~8% of cases) (see next section). Here, we summarize mouse models recapitulating other recurrent DLBCL-associated genetic lesions, including translocations of *BCL6*, loss-of-function mutations of *FBXO11* and *GNA13*, and a constellation of mutations targeting various components of the BCR, NF-κB, and terminal differentiation pathways, which represent a genetic hallmark of ABC-DLBCL.

### Disruption of the Gα13 Signaling Pathway

Almost one third of GCB-DLBCL (and ~58% of BL) carry deleterious mutations in multiple components of the Gα13 pathway, which is responsible for the confinement of GC B cells and also feeds the AKT pathway. The mutated genes include *GNA13* and, more rarely, *S1PR2* and *ARHGEF1* (138), indicating that this signaling cascade might be playing an important role across lymphoma subtypes. The observation that lower expression of S1PR2 is associated with worse survival in DLBCL (139) further supports its functioning as a tumor suppressor. Two conditional knock-out mouse models have been created, in which *Gna13* was either specifically deleted in GC B cells *via* crossing with the *Aicda-Cre* transgenic strain (140), or ablated in all B cells by using a mixed BM chimera approach (*Gna13^fl/fl^;mb1-Cre*) (138). Both models showed increased numbers of GC B cells with disordered GC architecture and altered DZ/LZ distribution, as well as higher levels of SHM activity and abnormal B-cell migration. Further supporting the critical role of the Gα13 pathway in lymphomagenesis, deletion of the *S1pr2* receptor led to the development of clonal B-cell lymphomas with morphologic, phenotypic and genetic characteristics resembling the human DLBCL in 50% of mice (141). Interestingly, lack of *Gna13* but not of *S1pr2* led to systemic dissemination of B cells in the lymph and blood, a finding that implies the existence of other G-protein coupled receptors regulating GC confinement. This observation led to the discovery of the P2RY8 receptor, which is also mutated in approximately 4% of GCB-DLBCL (138). Collectively, these studies provided insights into the mechanisms by which GNA13-deficient GC B cells leave the GC niche and spread systemically, and demonstrated a dual tumor-suppressor function for this signaling pathway *via* control of B-cell positioning and AKT activation.

### Modeling BCL6 Chromosomal Translocations

**BCL6** is a master regulator of the GC reaction and a common oncogene in both FL and DLBCL, where it constitutes a biological dependency. Deregulated expression of an intact BCL6 protein is induced in these tumors by a variety of genetic alterations that target the *BCL6* gene directly (e.g., chromosomal translocations or mutations in its 5’ non-coding sequences) (142–146) and indirectly (e.g. mutations of CREBBP, MEF2B, FBXO11) (95, 96, 147, 148). The endogenous *BCL6* promoter contains a number of regulatory elements that are bound by transcriptional repressors to downregulate its transcription at the exit from the GC (e.g. IRF4), or to maintain homeostatic levels in the GC *via* an autoregulatory negative feedback loop. These regulatory sequences are lost as a consequence of “promoter substitution” (cases with chromosomal translocations) or of point mutations, thus disrupting the BCL6 tightly restricted expression pattern (142–146). By sustaining constitutive BCL6 expression and/or activity, these lesions prevent the terminal differentiation of GC B cells, which remain stuck in a highly proliferative and genetically unstable environment potentially conducive to malignant transformation. One of the most common translocations found in FL and DLBCL, t(3;14)(q27;q32), was mimicked in the first GEMM recapitulating the genetics and biology of DLBCL (37). This model was created by knocking in an HA-tagged *BCL6* allele into the murine *IG* heavy chain locus, for expression under the endogenous *Iµ* promoter (*Iµ.HA.BCL6*). *Iµ.HA.BCL6* mice show GC hyperplasia with increased DZ : LZ ratio also in the absence of antigenic stimulation, and develop over time clonal lymphomas that recapitulate key aspects of DLBCL, most notably the evidence of AID-dependent aberrant somatic hypermutation and the presence of stochastic Myc-IgH translocations (37, 79). Interestingly, *BCL6* translocations can be found in both GCB- and ABC-DLBCL, but are enriched in a subset of ABC-DLBCL belonging to the BN2/C1 genetic subgroup, for which a marginal zone B cell origin has been postulated (29, 30). In this subgroup, *BCL6* deregulation frequently co-occurs with *NOTCH2*, *SPEN* or *TNAFAIP3* mutations, for which conditional mouse models have been generated (149–152). One possibility is thus that ectopic expression of *BCL6* induced by the translocation in marginal zone B cells cooperates with other “marginal zone” genes to ultimately cause this type of lymphoma. Although individually none of these other mutations was sufficient to drive lymphomagenesis, compound mice involving the *Iµ.HA.BCL6* model could shed light on the specific oncogenic events induced by the combined deregulation of BCL6 and NOTCH2 signaling (153).

### Biallelic Loss of *PRDM1/BLIMP1*

A distinctive feature of ABC-DLBCL, and particularly of the MCD/C5 genetic cluster, is the presence of genetic and epigenetic inactivation of the master plasma cell regulator **BLIMP1** (also known as PRDM1). In ~20% of cases, this is due to biallelic disruptive mutations and/or focal deletions of the *BLIMP1* locus, whereas in an additional subset of cases transcriptional silencing of BLIMP1 is achieved *via* deregulation of *BCL6* (154, 155). When engineered in the mouse, conditional B-cell specific *Blimp1* deletion (*Blimp1* ^fl/fl^; *Cd19-Cre* and *Blimp1* ^fl/fl^;*C*γ*1-Cre*) induced a block in plasma cell differentiation and the development of DLBCLs, the majority harboring somatically hypermutated *IG* genes. These tumors typically express IRF4 and CD138 and are negative for BCL6, a molecular pattern closer to the human ABC-DLBCL (156). As in other lymphoma models, the long latency and the clonality of the DLBCLs in *Blimp1* conditional KO animals indicate that oncogenic events affecting other pathways collaborate with BLIMP1 inactivation during lymphomagenesis. One important contributor to this process is the NF-κB transcription complex, which is constitutively active in virtually all ABC-DLBCLs and is targeted by genetic alterations at multiple levels in over half of the cases, frequently together with *BLIMP1* mutations (5, 157–160). Accordingly, DLBCLs developing in *Blimp1* conditional KO mice display nuclear active NF-κB (156), and a similar phenotype was reported in a conditional mouse model with combined disruption of *Blimp1* and enforced canonical NF-κB activation, obtained *via* a constitutively active IKK2 protein in GC B cells (*R26Stop* ^FL^ *Ikk2ca*;C*γ*1-Cre) (161).

### Constitutive Activation of the NF-κB Signaling Pathway

The canonical (RELA/p50 and c-REL/p50) and non-canonical (RELB/p52) NF-κB signaling pathways have been shown to play distinct roles in the GC response (162, 163). In most ABC-DLBCL cases, the activity of the canonical NF-κB transcription complex is sustained by the presence of genetic alterations affecting multiple genes that encode for positive or negative regulators of the BCR, CD40 receptor, and TLR signaling cascades, with the TLR adaptor protein MYD88 being mutated in over 30% of patient samples (157–160). Consistently, both *Cd19-Cre* driven and C*γ*1-Cre-driven expression of a ***Myd88^L252P^*** allele, corresponding to the most common activating mutation (L265P) in humans, promotes the occurrence of tumors that share several traits with the human ABC-DLBCL (164). *MYD88* mutations in the MCD/C5 ABC-DLBCL often occur in combination with *BCL2* copy number gains, and indeed, in the *Myd88^L252P^* mouse model, the combination with *Cd19-Cre* driven overexpression of BCL2 led to a significant increase in ABC-DLBCL-like B cell lymphomas (102). These tumors were sensitive to combination therapies with immune checkpoint blockade and BCL2 inhibition, revealing potentially actionable molecular vulnerabilities (102). In addition, a synergistic crosstalk was observed between the *Myd88^L252P^* hotspot mutation and ***CD79B*** mutations in a compound mouse model, exemplified by the accumulation of auto-reactive cells (165). Although these mice fail to develop overt lymphomas, their phenotype fits well with the suggested role of self-antigens in the survival of ABC-DLBCL cells *via* chronic activation of the BCR-signaling pathway (166). The *Myd88^L252P^* model may also provide a system to further dissect the signals emanating from a recently described multiprotein supercomplex formed by MYD88, TLR9 and the BCR (167).

In a smaller subset of human DLBCL, the observation of nuclear p52 translocation implies that the non-canonical NF-κB signaling cascade is also activated (157). Part of these cases can be explained by the presence of truncating mutations/deletions of the ***TRAF3*** gene, often coexisting with *BCL6* translocations. *TRAF3* encodes for a negative regulator of the NF-κB non-canonical pathway, involved in the degradation of the NF-κB inducing kinase (NIK). Accordingly, enforced expression of NIK and BCL6 in the GC, as obtained by conditional mutagenesis in the IμHABcl6;*NikstopFL*;*C*γ*1-Cre* mouse model, caused GC hyperplasia with blockade of terminal differentiation and development of IRF4-positive DLBCL (168). Notably, *NikstopFL*;*C*γ*1-Cre* mice display overt plasma cell hyperplasia but do not succumb to tumors; thus, the oncogenic function of the alternative NF-κB pathway may require the concomitant disruption of terminal B-cell differentiation, which in this case was achieved by deregulated *BCL6* expression. An analogous synergistic phenotype was observed by combining constitutive NF-κB activation and Blimp1 loss in the compound *Blimp1* ^fl/fl^;*R26Stop* ^FL^ *Ikk2ca*;*C*γ*1-Cre* model (161).

### Deletion of FBXO11

F-box protein 11 (**FBXO11**) is a member of the F-box protein family that functions in the protein degradation pathway. FBXO11 is a subunit of the substrate-recognition complex SKP1-cullin-1-F-box-protein (SCF) E3 ligase, which leads to ubiquitylation and degradation of numerous target proteins, including BCL6 and BLIMP1 (147, 169, 170). In DLBCL, *FBXO11* monoallelic mutations and/or deletions are present in 6% of cases and correlate with increased BCL6 expression (147). To recapitulate these events, a conditional *Fbxo11* knock-out mouse model was crossed with the GC specific *C*γ*1-Cre* driver, documenting a direct link between Fbxo11 loss and the formation of enlarged GCs with increased BCL6 protein levels in response to antigenic challenge (148). Aged *Fbxo11*-deleted mice, when chronically immunized, develop various B-cell lymphoproliferative phenotypes including a low frequency of overt DLBCL. The low tumor penetrance indicates that additional alterations are required for full transformation, along with *FBXO11* inactivation. Nonetheless, this model confirmed a tumor-suppressor role for *FBXO11* in lymphomagenesis, and could be utilized to gain further insights into the mechanism underlying the pathogenetic process.

## The Challenge of Double Hit Lymphomas and tFL

High-grade large B cell lymphomas with concurrent MYC and BCL2 (or BCL6) translocations, previously known as double-hit (DHL)/triple-hit lymphomas, represent a rare category of tumors that is now recognized as a separate provisional entity in the revised WHO classification (3). DHLs typically display a GCB-like phenotype, different from tumors where these two genes are co-expressed in the absence of genetic alterations (171), and, although rare, constitute an area of intense research due to their poor clinical outcome, even though more recent studies suggest a certain degree of heterogeneity, with cases showing a more favorable prognosis (3, 172, 173). MYC translocations are also seen as a secondary genetic alteration occurring on a BCL2-rearranged genetic background during histologic transformation of FL to DLBCL, an adverse event denoted by an aggressive clinical course (85). As such, a faithful model recapitulating the genetics and phenotype of DHLs or tFL would be an invaluable tool for uncovering potential vulnerabilities and pre-clinically testing novel therapeutic principles. Efforts to understand the co-operation between BCL2 and MYC *in-vivo* have been conducted, for instance in transgenic mice expressing these two genes under the control of the Eµ enhancer (174, 175). However, the early timing of MYC deregulation invariably leads to the clonal expansion of immature B cells. Thus, the construction of GEMMs that faithfully mimic the genetics and the pathobiology of these conditions with regard to both the developmental stage at which the translocations take place (for MYC, a GC B cell undergoing SHM or CSR) and the GC origin of the developing tumors (i.e., somatically mutated *IGHV* genes and immunophenotypic markers of GC B cells) remains a gap in the field.

## Concluding Remarks

GEMMs have revolutionized the study of cancer biology and will remain an invaluable tool in biomedical research, by allowing to elucidate the *in vivo* consequences of novel mutational targets (including coding and non-coding regions of the genome), study the mechanisms underlying the development of B cell lymphomas, and test new therapeutic modalities in a pre-clinical setting. However, no single model can fully reproduce the complexity of the human tumors, which evolve through the sequential acquisition of multiple genetic and epigenetic changes, in concert with an adaptive microenvironment. Investigating the synergistic interactions that are implicated in the malignant transformation process and the plethora of novel therapeutic agents that are being considered for pre-clinical testing warrants the need for more rapid, high-throughput, and possibly less expensive approaches to modeling cancer. While the generation of lymphoma organoids, the expansion of PDX repositories, and the advent of increasingly sophisticated approaches such as the CRISPR-Cas9 editing technique may help to overcome some of the limitations, the judicious construction and study of GEMMs will likely continue to deliver advances that can greatly contribute to improving the management of B cell malignancies.

## Author Contributions

SM and SK wrote the first draft of the manuscript. LP supervised and finalized the work. All authors contributed to the article and approved the submitted version.

## Funding

This work was supported in part by grants R01-CA172492 (to LP) and a Leukemia & Lymphoma Society Translational Research Project award (LP).

## Conflict of Interest

The authors declare that the research was conducted in the absence of any commercial or financial relationships that could be construed as a potential conflict of interest.

## Publisher’s Note

All claims expressed in this article are solely those of the authors and do not necessarily represent those of their affiliated organizations, or those of the publisher, the editors and the reviewers. Any product that may be evaluated in this article, or claim that may be made by its manufacturer, is not guaranteed or endorsed by the publisher.
