# Supplementary material for: Mouse Models of Germinal Center Derived B-Cell Lymphomas
Source: Front Immunol. 2021 Aug 12;12:710711. doi: 10.3389/fimmu.2021.710711 (PMC8387591; doi:10.3389/fimmu.2021.710711)
Supplement: Supplementary file 1 [file Table_1.docx]

**Table 1.** GEMMs recapitulating genetic changes associated with human GC B cell lymphomas

| **Gene** | **Lymphoma** | **Mutation Type** | **Mouse Model** | **Approach** | **Target cell** | **Phenotype** | **Ref** |
| --- | --- | --- | --- | --- | --- | --- | --- |
| *Myc/PI3K* | BL | deregulated expression + gain of function mutation | *R26Stop*^FL^*Myc*;*R26Stop*^FL^*P100**;Cγ1-Cre | cKI | GC B cells | BL | Sander et al. 2012 |
| *Bcl2* | FL, GCB-DLBCL | deregulated expression | VavP-*Bcl2* | transgene insertion | HPC | FL | Egle et al. 2004 |
|  |  |  | *Bcl2*-Ig | transgene insertion | B cells | FL | McDonnell et al. 1989 |
|  |  |  | *BCL2^tracer^* | TI/adoptive transfer | B cells | FL | Sungalee et al. 2014 |
| *Kmt2d^* | FL, DLBCL | genetic deletion | *Kmt2d*^fl/fl^;VavP-*Bcl2*;Cγ1-Cre | cKO | GC B cells | FL, DLBCL | Zhang et al. 2015 |
|  |  |  | *Kmt2d*^fl/fl^;VavP-*Bcl2*;Cd19-Cre | cKO | B cells | FL, DLBCL | Zhang et al. 2015 |
| *Crebbp^* | FL, DLBCL | genetic deletion | *Crebbp*^fl/fl^;VavP-*Bcl2*;Cγ1-Cre | cKO | GC B cells | FL | Zhang et al. 2017 |
|  |  |  | *Crebbp*^fl/fl^;VavP-*Bcl2*;Cd19-Cre | cKO | B cells | FL | Zhang et al. 2017 |
|  |  |  | *Crebbp*^fl/fl^;Eµ-*Bcl2*; Mb1-Cre | cKO | B cells | FL, DLBCL | Garcia Ramirez et al. 2017 |
| *H1e/H1c^* | FL, DLBCL | genetic deletion | *H1c^-/+^H1e^-/+^;VavP-Bcl2* | cKO | All cells | FL, DLBCL | Yusufova et al, 2021 |
| *Ezh2^* | FL, GCB-DLBCL | gain of function mutation | *Ezh2*^Y641F/+^;IµHABCL6;Cγ1-Cre | cKI/adoptive transfer | GC B cells | DLBCL | Beguelin et al. 2016 |
|  |  |  | *Ezh2*^Y641F/+^;VavP-Bcl2;Cγ1-Cre | cKI/adoptive transfer | GC B cells | FL, DLBCL | Ennishi et al., 2018 |
| *Mef2b* | FL, GCB-DLBCL | gain of function mutation | Mef2b^D83V/+^;CD21-Cre | cKI | GC B cells* | FL, DLBCL | Brescia et al. 2018 |
|  |  |  | Mef2b^D83V/+^;BCL2-Ig;CD21-Cre | cKI | GC B cells* | FL, DLBCL | Brescia et al. 2018 |
| *Rragc* | FL | gain of function mutation | Rragc^S74C/+^ or Rragc^T89N/+^;VavP-Bcl2 | TI/adoptive transfer | All cells | FL | Ortega-Molina et al. 2019 |
| Gna13 | GCB-DLBCL, BL | genetic deletion | Gna13^fl/fl^;Mb1-Cre | cKO/adoptive transfer | GC B cells** | GC BCL | Muppidi et al. 2014 |
|  |  |  | Gna13^fl/fl^;R26Stop^FL^Myc;Aid-Cre | cKO | GC B cells | GC BCL | Healy et al. 2016 |
| *Bcl6* | DLBCL | deregulated expression | IµHABCL6 | KI | GC B cells | DLBCL | Cattoretti et al. 2009 |
| *Prdm1* | ABC-DLBCL | genetic deletion | Blimp1^fl/fl^;Cγ1-Cre | cKO | GC B cells | DLBCL | Mandelbaum et al. 2010 |
|  |  |  | Blimp1^fl/fl^;R26Stop^FL^Ikk2ca; Cγ1-Cre | cKO | GC B cells | DLBCL | Calado et al. 2010 |
| *Fbxo11* | DLBCL | genetic deletion | Fbxo11^fl/fl^;Cγ1-Cre | cKO | GC B cells | DLBCL | Schneider et al, 2016 |
| *Myd88* | ABC-DLBCL | gain of function mutation | Myd88^p.L265P/+^;Aid-Cre | cKI | GC B cells | LPD, DLBCL | Knittel et al. 2016 |
|  | ABC-DLBCL |  | Myd88^p.L265P/+^;CD19-Cre;Rosa26LSL.BCL2 | cKI | B cells | LPD, DLBCL | Flumann et al., 2021 |
| *Tet2* | FL, DLBCL | genetic deletion | Tet2^fl/fl^;VavP-Cre | cKO | HPC | B cell lymphomas | Dominguez et al. 2018 |
|  |  |  | Tet2^fl/fl^;IµHABCL6;Cγ1-Cre | cKO/adoptive transfer | GC B cells | B cell lymphomas | Dominguez et al. 2018 |

Abbreviations: TI, targeted insertion; KO, knock-out; cKO, conditional KO; KI, knock-in; HPC, hematopoeietic progenitor cell; GC BCL, GC-derived B cell lymphoma; LPD, lymphoproliferative disorder.

* Cre-mediated recombination in mature B cells, but the endogenous promoter is activated in GC B cells.

** Cre-mediated recombination in all B cells, but the endogenous promoter is upregulated in GC B cells.

^ GC-derived lymphomas observed only in cooperation with Bcl2 deregulated expression.

Mouse models obtained by adoptive transfer of retrovirally transduced HPCs not included.
